# Greater neighborhood deprivation is related to shorter survival in behavioral‐variant frontotemporal degeneration

**DOI:** 10.1002/alz.095411

**Published:** 2025-01-09

**Authors:** Rory Boyle, Nadia Dehghani, Shana D. Stites, Brian Nelson, David J Irwin, Corey T McMillan, Lauren Massimo

**Affiliations:** ^1^ Penn Frontotemporal Degeneration Center, Department of Neurology, Perelman School of Medicine, University of Pennsylvania, Philadelphia, PA USA; ^2^ Department of Psychiatry, Perelman School of Medicine, University of Pennsylvania, Philadelphia, PA USA

## Abstract

**Background:**

Emerging evidence relates neighborhood deprivation to cognitive decline and neurodegeneration but this is understudied in frontotemporal degeneration (FTD). We assessed the association of neighborhood deprivation with survival in 147 individuals with behavioral‐variant FTD (bvFTD).

**Method:**

Neighborhood deprivation was measured using the Area Deprivation Index (ADI), a national percentile ranking of deprivation based on income, education, employment, and housing quality. ADI was split into tertiles: lowest deprivation (ADI:1‐19), intermediate deprivation (ADI:20‐40), and highest deprivation (ADI:41‐93). In a Cox regression, we examined the association between ADI (reference group = lowest deprivation) and survival time (time from symptom onset to death), adjusting for age at symptom onset, sex (reference group = women), and education years. Individuals alive at study close were censored at their last clinic visit.

**Result:**

Older age at symptom onset was associated with shorter survival (hazard ratio [HR] = 1.03[95%CI = 1.01–1.06], p = 0.005). Male sex was associated with longer survival (HR = 0.63[95%CI = 0.42–0.96], p = 0.03). Highest deprivation (HR = 1.84[95%CI = 1.09‐3.1], median survival years = 7[women]‐9[men], p = 0.017), but not intermediate deprivation (HR = 1.58[95%CI = 0.95‐2.63], median survival years = 7[women]‐10[men], p = 0.081), was associated with shorter survival time compared to the lowest deprivation group (median survival years = 10[women]‐12[men]). Similar results were observed in a sex‐stratified model.

**Conclusion:**

Our results suggest that increased neighborhood deprivation contributes to shorter survival in bvFTD. Further research is needed to replicate this finding and uncover the underlying mechanisms.

